# Carbon Nanostructures Derived through Hypergolic Reaction of Conductive Polymers with Fuming Nitric Acid at Ambient Conditions

**DOI:** 10.3390/molecules26061595

**Published:** 2021-03-13

**Authors:** Nikolaos Chalmpes, Dimitrios Moschovas, Iosif Tantis, Athanasios B. Bourlinos, Aristides Bakandritsos, Renia Fotiadou, Michaela Patila, Haralambos Stamatis, Apostolos Avgeropoulos, Michael A. Karakassides, Dimitrios Gournis

**Affiliations:** 1 Department of Materials Science & Engineering, University of Ioannina, 45110 Ioannina, Greece; chalmpesnikos@gmail.com (N.C.); dmoschov@uoi.gr (D.M.); aavger@uoi.gr (A.A.); mkarakas@uoi.gr (M.A.K.); 2 Regional Centre of Advanced Technologies and Materials, Czech Advanced Technology and Research Institute, Palacký University Olomouc, Křížkovského 511/8, 779 00 Olomouc, Czech Republic; iosif.tantis@upol.cz; 3 Physics Department, University of Ioannina, 45110 Ioannina, Greece; 4 Regional Centre of Advanced Technologies and Materials, Palacky University in Olomouc, Slechtitelu 27, 779 00 Olomouc, Czech Republic; a.bakandritsos@upol.cz; 5 Nanotechnology Centre, VŠB–Technical University of Ostrava, 17. listopadu 2172/15, 708 00 Ostrava-Poruba, Czech Republic; 6 Biotechnology Laboratory, Department of Biological Applications and Technologies, University of Ioannina, 45110 Ioannina, Greece; renia.fotiadou@gmail.com (R.F.); michaelapatila@gmail.com (M.P.); hstamati@uoi.gr (H.S.)

**Keywords:** conductive polymers, fuming nitric acid, hypergolics, carbon nanostructures, ambient conditions, rocket fuels

## Abstract

Hypergolic systems rely on organic fuel and a powerful oxidizer that spontaneously ignites upon contact without any external ignition source. Although their main utilization pertains to rocket fuels and propellants, it is only recently that hypergolics has been established from our group as a new general method for the synthesis of different morphologies of carbon nanostructures depending on the hypergolic pair (organic fuel-oxidizer). In search of new pairs, the hypergolic mixture described here contains polyaniline as the organic source of carbon and fuming nitric acid as strong oxidizer. Specifically, the two reagents react rapidly and spontaneously upon contact at ambient conditions to afford carbon nanosheets. Further liquid-phase exfoliation of the nanosheets in dimethylformamide results in dispersed single layers exhibiting strong Tyndall effect. The method can be extended to other conductive polymers, such as polythiophene and polypyrrole, leading to the formation of different type carbon nanostructures (e.g., photolumincent carbon dots). Apart from being a new synthesis pathway towards carbon nanomaterials and a new type of reaction for conductive polymers, the present hypergolic pairs also provide a novel set of rocket bipropellants based on conductive polymers.

## 1. Introduction

Polyaniline (PANI) is a unique conductive polymer that has attracted considerable attention due to its fascinating electrical conductivity and diverse chemistry [1,2,3,4]. Although its monomer aniline is widely known as a hypergolic propellant [5], no report in the literature refers to polyaniline for a similar purpose. Since polyaniline and aniline share a common chemical structure, it is expected that the polymer will act similarly to the monomer in terms of hypergolicity. A certain advantage of polyaniline as hypergolic fuel compared to liquid aniline is the solid-state matter of the polymer (e.g., solid fuels, due to their higher density, tend to occupy less space than liquid ones). In addition, the non-volatile nature of the solid polymer makes it safer and a much better carbon precursor than the volatile liquid monomer (e.g., polymers tend to provide higher carbon yields than monomers upon carbonization). Characteristically, the ignition of aniline by fuming nitric acid results in a minor carbon residue (<1% after thorough washings) for practical consideration in this context [6].

Carbon constitutes a cornerstone in material science due to its variety of forms and outstanding properties [7]. It is generally produced by several techniques such as pyrolysis of organic compounds [8], hydrothermal synthesis [9] or chemical vapor deposition methods (CVD) [10]. However, all these methods require high temperature and hence energy consumption in order to achieve carbonization. In this respect, carbon synthesis at ambient conditions is an energy-saving manner that remains a challenge.

Hypergolics appear to be an interesting option in this direction. In hypergolics an organic fuel and a strong oxidizer ignite spontaneously upon contact at room temperature and atmospheric pressure. As a result, hypergolics find massive applications in rocket fuels and propellants. But this same concept, when applied at a smaller scale, also provides a new synthetic approach towards the preparation of diverse carbon nanostructures at ambient conditions in an exothermic manner. Scaling-up the method under safer conditions is a crucial factor for the proposed technique to be widely accepted in the future. However, it is worth mentioning that several other preparative methods posing serious hazards, such as flame spray pyrolysis, are widely used today due to technical advancements over time. The ever-increasing demands in rocket fuel engineering will probably determine the path towards the development of pilot apparatus for large-scale hypergolic synthesis.

Recently, our group has introduced hypergolic reactions as a novel synthesis tool towards the rapid and spontaneous preparation of a large variety of functional carbon nanomaterials at ambient conditions. For example, carbon nanosheets were previously obtained from self-ignition of lithium dialkylamides in air [11], highly crystalline graphite through the spontaneous reaction of the acetylene-chlorine hypergolic mixture [12], carbon dots or nanosheets via hypergolic pairs based on Girard’s reagent T or nitrile rubber and fuming nitric acid as strong oxidizer [6], carbon nanosheets or hydroxylated fullerenes using coffee grains or C_60_ as carbon sources and sodium peroxide as strong oxidizer [13], dense or hollow spheres derived from the reaction of ferrocene with liquid bromine at room temperature [14], carbon nanodiscs obtained through the cyclopentadienyllithium-fuming nitric acid hypergolic pair [15] and, lastly, carbon nanosheets via the hypergolic reaction of furfuryl alcohol with fuming nitric acid at ambient conditions [16]. In the majority of the reactions the released energy was converted into useful chemical [11], thermoelectric [6], photovoltaic [13] or mechanical [16] work. Interestingly, the nitrile rubber case aptly shows a practical way to convert polymer waste into useful material through hypergolics [6]. Moreover, the obtained carbon nanostructures were successfully utilized in hexavalent chromium removal, decolorization of water-soluble organic dyes as well as graphene conductive inks [12].

In search of new pairs, herein we present for the first time the hypergolic ignition of polyaniline organic fuel by fuming HNO_3_ at ambient conditions towards the formation of carbon nanosheets. Single layers can be further produced from the nanosheets using the liquid-phase exfoliation technique. In order to show the general character of the method, we equally apply the concept to other conductive polymers (e.g., polythiophene, polypyrrole) towards the isolation of different type carbon nanostructures, such as photoluminescent carbon dots. Overall, the discussed reactions are new in the field of conductive polymers, offering novel footpaths to nanocarbon synthesis as well as new solid propellants for rocket fuel applications. Although conductive polymers are considered expensive reagents, it should be emphasized that their relatively high cost is equivalent to or even less than the forfeit of ionic liquids, the latter being widely studied as hypergolic fuels and propellants [17,18,19,20,21].

## 2. Results and Discussion

### 2.1. Polyaniline-HNO_3_ Hypergolic Pair

**Figure 1:**
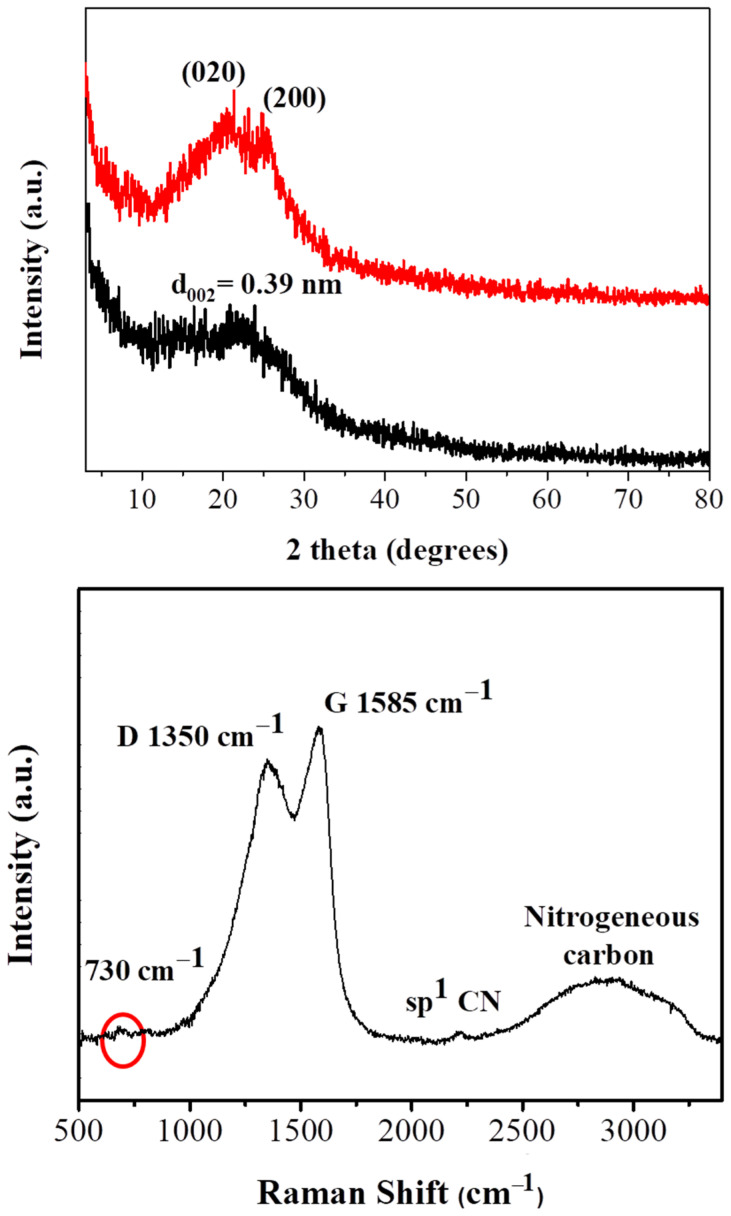
X-ray diffraction (XRD) pattern (top black line) and Raman spectrum (bottom) of the carbon sample. The XRD pattern of PANI is also presented for comparison (top red line).

Carbon formation after ignition of polyaniline by fuming nitric acid is evidenced by powder X-ray diffraction (XRD) diffraction experiments. In respect to the starting polymer, the characteristic diffraction peaks at 20.4° and 25° (Figure 1 top, red line) corresponding to the (020) and (200) crystal planes of PANI [22,23] are present. These peaks are attributed to π–π interchain stacking, as well as, to the parallel and perpendicular periodicity of the polymer chains [24]. On the contrary, the XRD pattern of the derived carbon exhibits a very broad reflection centered at d_002_ = 3.9 Å (Figure 1 top, black line). This value is higher than the interlayer distance of crystalline graphite (3.34 Å) [12], indicating the formation of highly disordered carbon [25].

Raman spectroscopy (Figure 1, bottom) reveals the typical for carbon materials G (1585 cm^–1^) and D (1350 cm^−1^) bands [26]. The presence of these peaks implies the formation of amorphous carbon containing both sp^3^/sp^2^ domains (the intensity ratio of the two bands is estimated as I_D_/I_G_ ~ 0.8). The carbonized solid additionally displays a strong yet broad band at 2900 cm^–1^ and a weak peak at 730 cm^−1^, indicative of nitrogen incorporation into the carbon lattice [27]. The weak peak near 2200 cm^−1^ is ascribed to sp^1^ C≡N stretching bonds [28].

**Figure 2:**
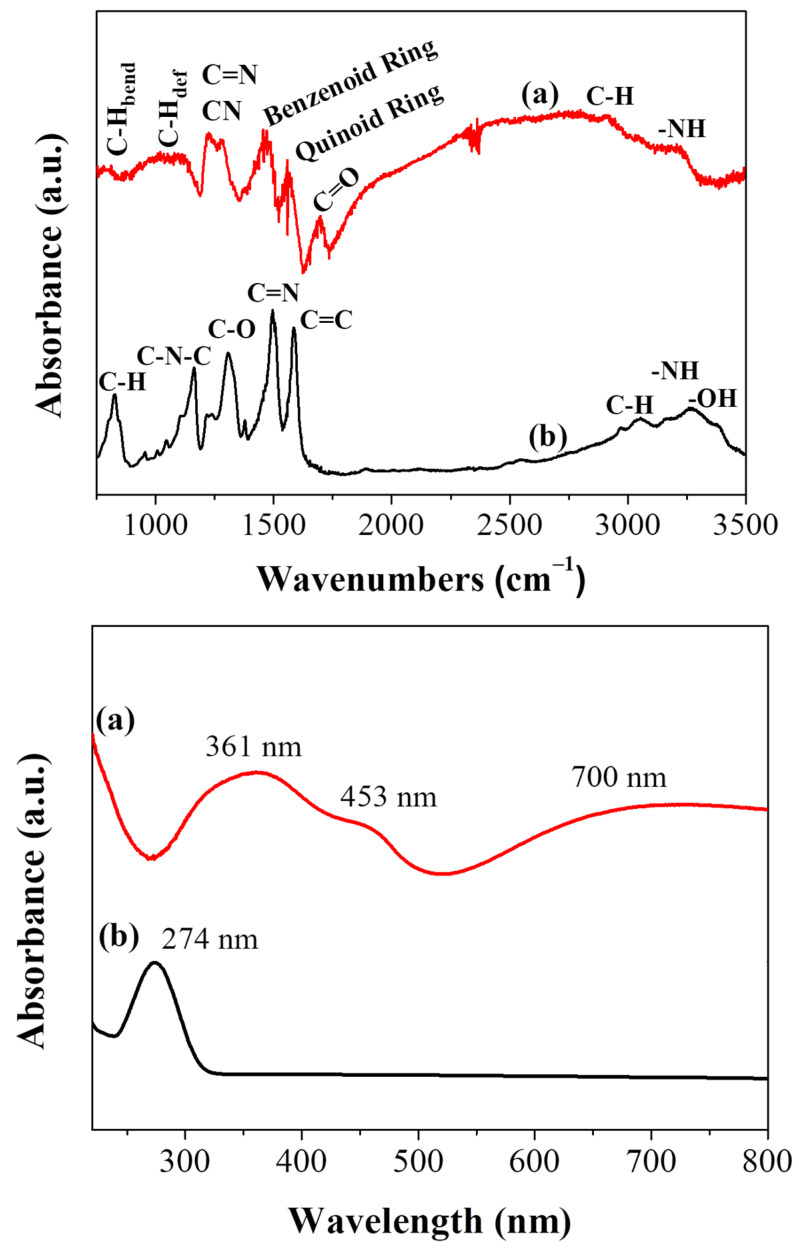
ATR-IR spectra (**top**) and UV-vis spectra (**bottom**) of (**a**) PANI and (**b**) carbon nanosheets.

The attenuated total reflectance -infrared spectrum (ATR-IR) of the carbon solid compared to that of the starting polyaniline is shown in Figure 2, top. As far as PANI is concerned, the peaks at 1572 cm^−1^ and 1494 cm^−1^ are assigned to the stretching deformation mode of the quinoid and benzenoid rings, respectively [24,29]. The carbonyl group of the acetic acid used for the polymer treatment is clearly distinguished at 1700 cm^−1^. The bands at 1280 cm^–1^ and 1226 cm^−1^ are attributed to C–N and C=N stretching vibrations, the weak shoulder at 1080 cm^−1^ to aromatic C–H in plane deformation and the weak band at 780 cm^−1^ to the out of plane bending mode of aromatic C–H groups. The weak shoulder near 3000 cm^−1^ is assigned to C–H moieties while the peak at 3250 cm^−1^ is attributed to the stretching vibrations of N–H moieties [24,29,30,31]. On the other hand, the spectrum of the polyaniline-derived solid is suggestive of oxidized carbon decorated with oxygen-containing functionalities and incorporating nitrogen in its structure (Figure 2, top). The intense bands at 1583 cm^−1^ and 1496 cm^−1^ originate from the C=C and C=N stretching vibrations, respectively, whereas the band at 1307 cm^−1^ from the C–O vibration. The peak at 1163 cm^−1^ is correlated with the C–N–C bending vibration. The material additionally contains aliphatic/aromatic hydrogens as evidenced by the stretching (3000 cm^−1^) and bending (825 cm^−1^) modes of C–H group along with the stretching vibrations of N–H, –OH in the high region frequencies of 3200–3400 cm^−1^ [32,33].

The Ultraviolet–visible (UV-vis) spectrum of the carbon nanosheets compared to polyaniline is shown in Figure 2, bottom. Both materials were suspended in ethanol via sonication prior optical measurements. The absorption peak of polyaniline at 361 nm is attributed to a π–π* transition of the benzene ring system, whereas those at 453 and 700 nm are assigned to the polymer’s polaron–π* and *π*–polaron transitions, respectively [34,35]. In contrast, the UV–vis spectrum of the nanosheets exhibits only one well-discernable peak at 274 nm ascribed to π-π* transitions within long-ranged sp^2^ domains (Figure 2, bottom) [36].

**Figure 3:**
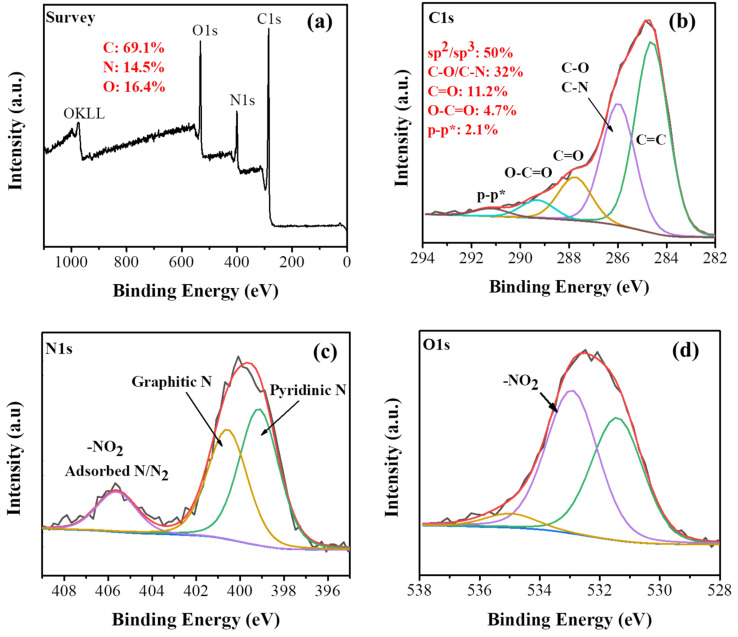
(**a**) X-ray photoelectron spectroscopy (XPS) survey spectrum, (**b**) C1s, (**c**) N1s and (**d**) O1s deconvoluted high resolution spectra of the carbon solid.

The X-ray photoelectron spectroscopy (XPS) survey spectrum of the carbon material clearly reveals the presence of C (69.1%), O (16.4%) and N (14.5%) in the sample (Figure 3a). The high-resolution C1s spectrum shows a peak at a binding energy of 285.9 eV due to C−N and C−O bonds (Figure 3b). The other C1s components are attributed to sp^2^/sp^3^ carbons (284.6 eV, highest relative area), C=O (287.8 eV) and O−C=O (289.3 eV). The satellite component at 291.2 eV is ascribed to π–π* transitions within sp^2^ conjugated carbons [37]. The high resolution Ν1s spectrum was deconvoluted into three components (Figure 3c). The main component at 399.1 eV with 47.5% area is attributed to the pyridinic N while the peak at 400.6 eV corresponds to graphitic N [38]. Finally, the component at 405.5 eV (14%) can be ascribed to both adsorbed N/N_2_ and the presence of −NO_2_ groups (planar and tilted −NO_2_) [39,40]. The presence of −NO_2_ groups is further evidenced in the O1s high resolution XPS spectrum (Figure 3d) by the characteristic peak at 532.9 eV [41]. The high area of the C−N species at C1s alongside with the graphitic and pyridinic components at N1s verifies that nitrogen is mainly covalently bonded in the carbon matrix.

**Figure 4:**
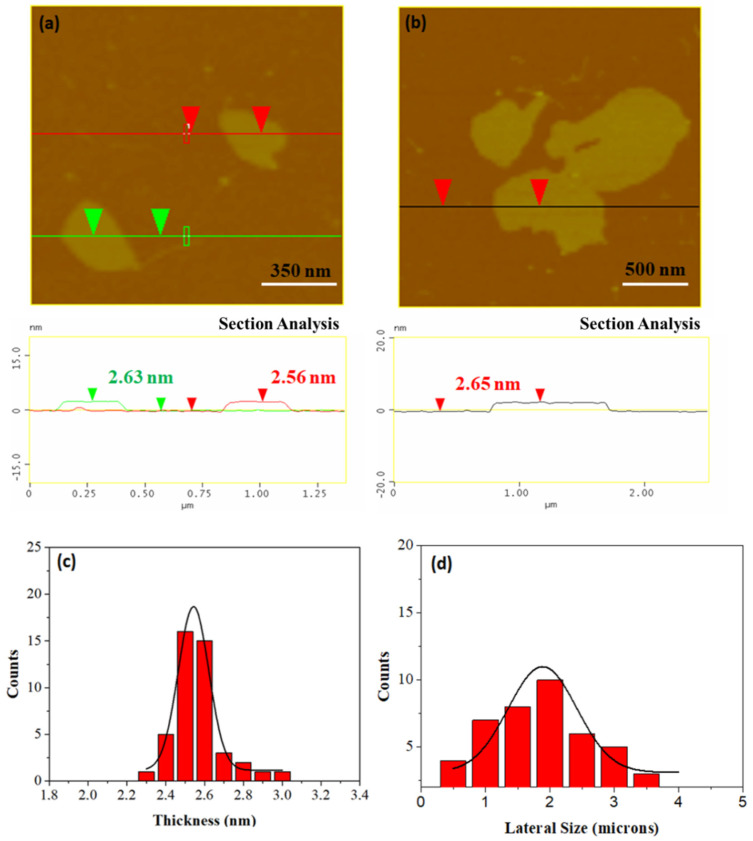
(**a**,**b**) Atomic force microscopy (AFM) images of cross-sectional analysis of selected carbon nanosheets. (**c**,**d**) Statistical analysis histograms of thickness and lateral size of 43 randomly selected sheets.

The average thickness of the carbon nanosheets as evaluated by atomic force microscopy (AFM) cross sectional images is close to 2.5–2.6 nm (Figure 4a,b). To properly investigate nanosheets size distributions, we performed a statistical analysis based on AFM representative imaging. An amount of 43 randomly selected carbon nanostructures were recorded and used for the statistical analysis histograms. The results obtained are shown in Figure 4c,d images. The thickness histogram shows that the Gaussian curve fit is centered on an average value of 2.6 nm. On the other hand, the lateral size distribution covers a wide range in the micron scale with an average value of 2 μm.

**Figure 5:**
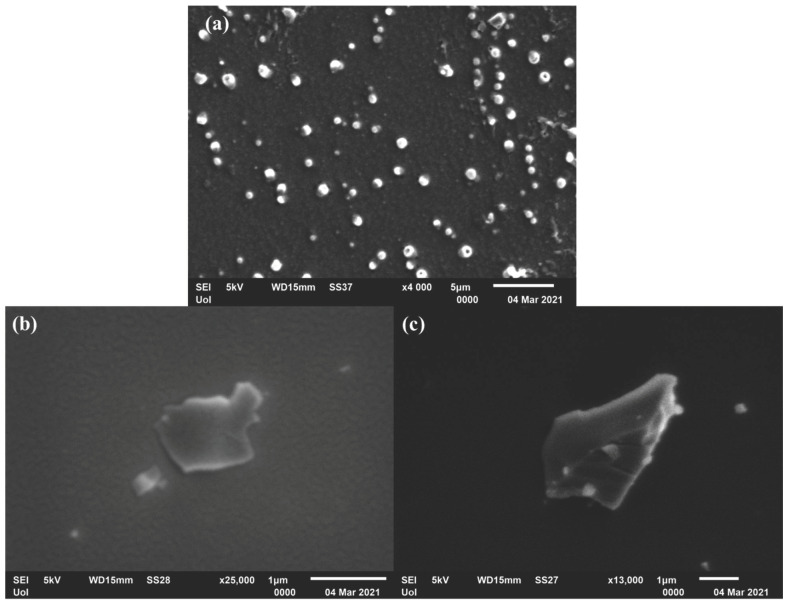
Scanning electron microscopy (SEM) images of (**a**) PANI and (**b**,**c**) carbon nanosheets.

**Figure 6:**
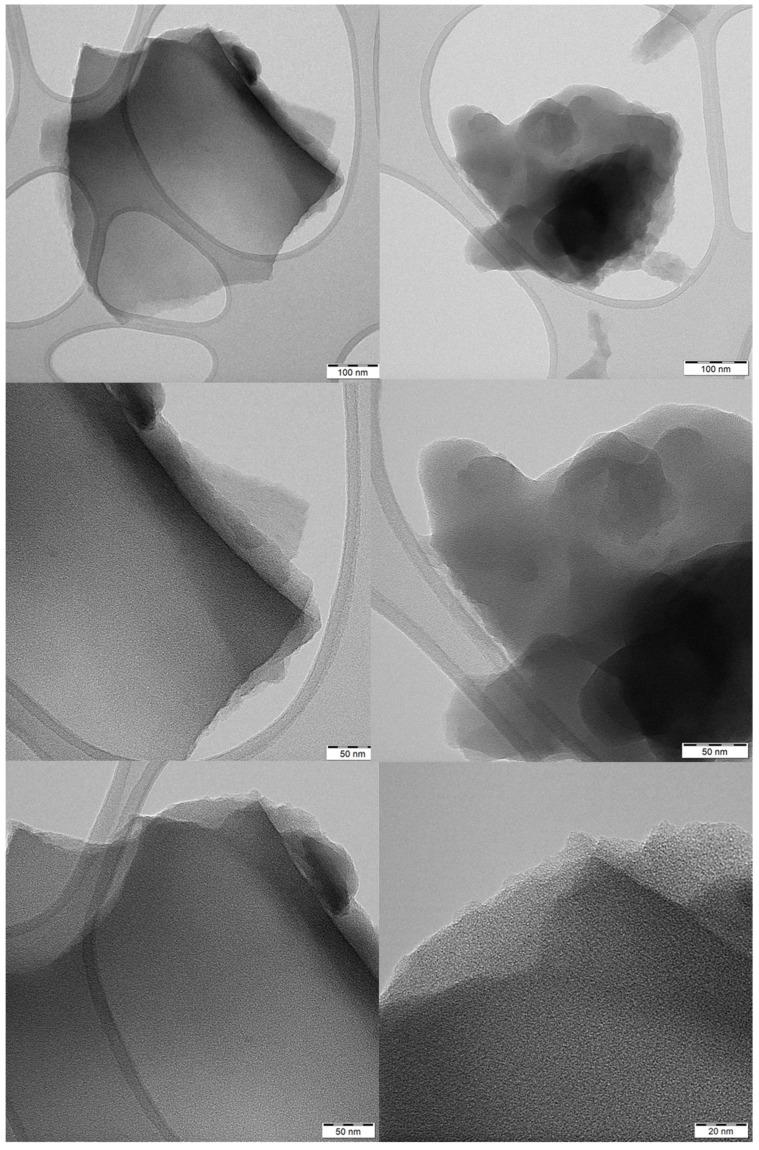
Representative transmission electron microscopy (TEM) micrographs of carbon nanosheets. The micrographs show some individual sheets along with their edge morphology.

The morphological characteristics of the derived carbon were also studied in detail using electron microscopies. Scanning electron microscopy (SEM) and transmission electron microscopy (TEM) images reveal exclusively the presence of carbon nanosheets in the studied sample (Figure 5 and Figure 6). The nanosheets appear compact possessing micron-sized lateral dimensions. In addition, they display multilayer structure near the edges due to stacks of individual layers within a nanosheet (Figure 6). It should be highlighted that the morphology of the starting PANI is radically different based on SEM examination (Figure 5), clearly showing the presence of globular nanoparticles.

**Figure 7:**
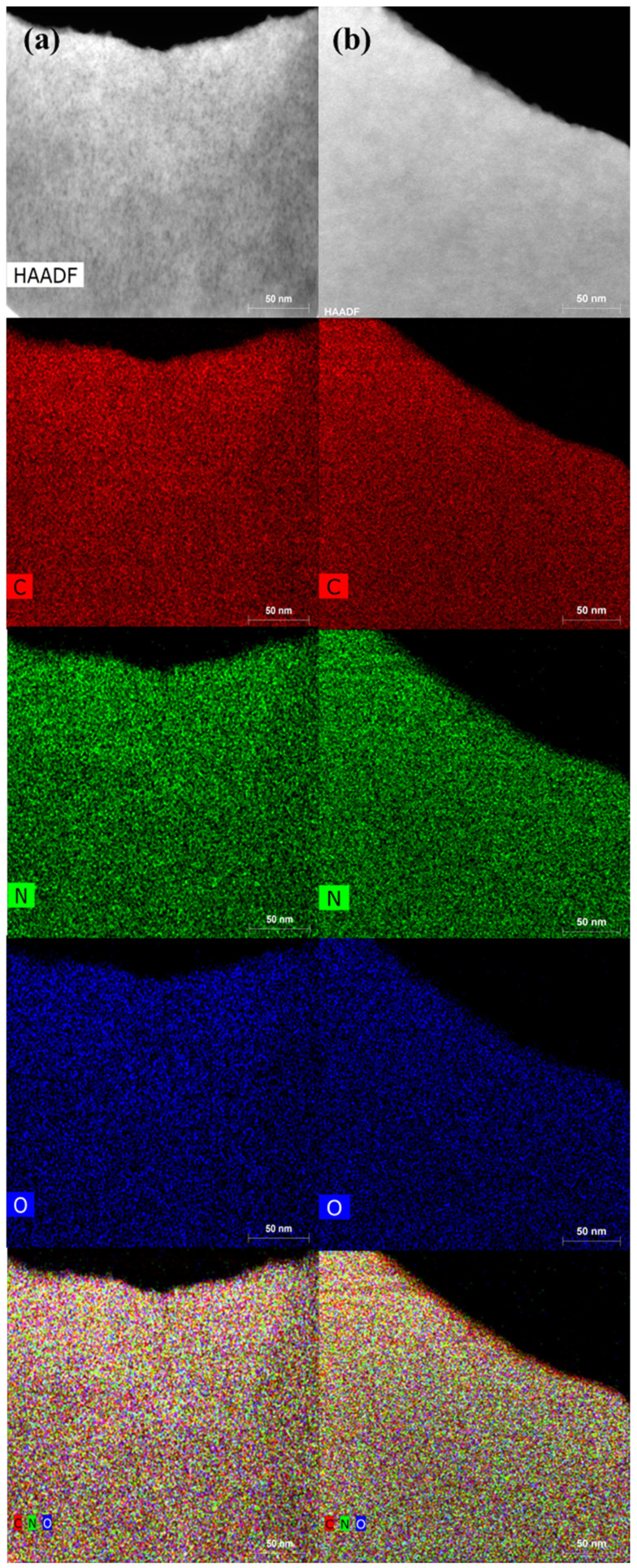
High-resolution transmission electron microscopy (HR-TEM) chemical mapping of carbon nanosheets (**a**,**b**). Red color denotes C, green N and blue O. The images at the bottom show the uniform distribution of C, N and O within the sheets.

High-resolution transmission electron microscopy (HRTEM) chemical mapping shows an even distribution of the XPS-detected C, N, and O elements in the sample (Figure 7). All elements are uniformly distributed over the surface of the carbon matrix, thus ensuring a homogeneous composition within the sample.

**Figure 8:**
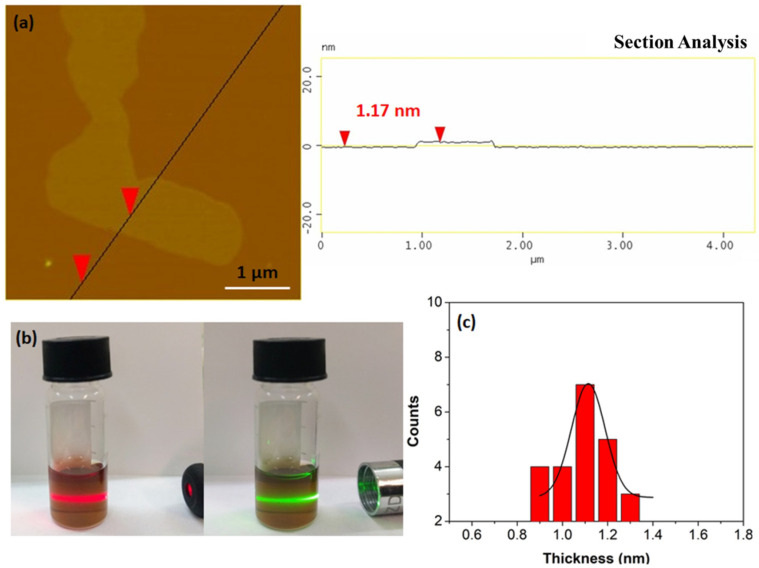
(**a**) Atomic force microscopy (AFM) height profile of a single sheet. (**b**) The colloidal dispersion of the single sheets in DMF exhibiting a strong Tyndall effect using a red or green laser pointer. (**c**) AFM statistical analysis of thickness for 25 randomly selected nanosheets.

Furthermore, it is possible to produce single sheets from the flakes using the liquid-phase exfoliation technique [42]. To this aim, 50 mg of the carbon solid were suspended via 3 h sonication (160 W) in 12.5 mL dimethylformamide (DMF) in a sealed glass vial. The suspension was left to rest for 3 days in order to settle down any solid particulates and the supernatant was collected as a clear colloid giving a strong Tyndall effect using a green or red laser pointer (Figure 8b). AFM studies clearly reveal the presence of single sheets in the colloid (Figure 8a). A statistical analysis of thickness was also made for comparison reasons. Accordingly, the dispersed sheets exhibit thickness of 0.9–1.3 nm, that is, less than the average thickness of the starting flakes (2.6 nm) (Figure 8c).

### 2.2. Application of the Method to Other Conductive Polymers

**Figure 9:**
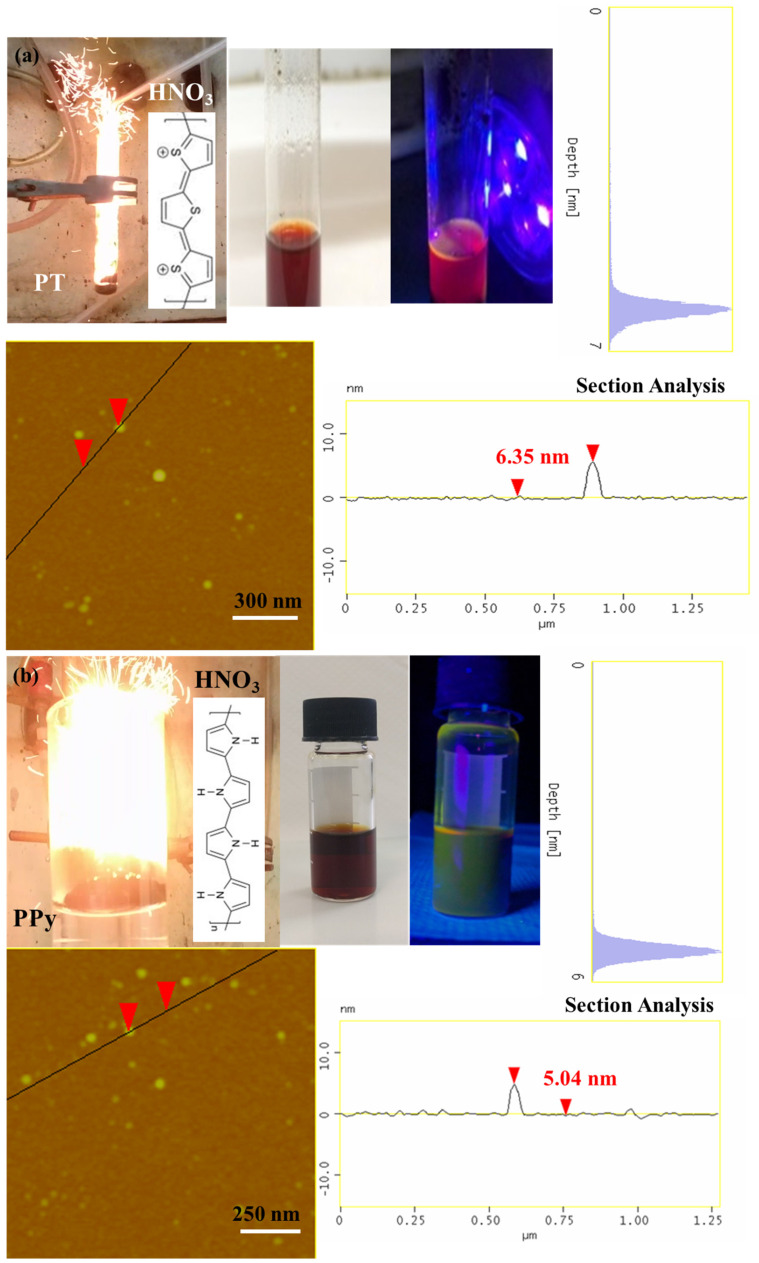
Hypergolic ignition of PT (**a**) and PPy (**b**) by fuming HNO_3_ results in photoluminescent carbon dots (see for instance the corresponding acetone dispersions under natural and UV light). The AFM topography images of (**a**) PT- and (**b**) PPy-derived dots along with their section-analysis and depth-analysis histograms are also included.

It is interesting to note that the method can be successfully extended to other conductive polymers, beyond polyaniline, towards the preparation of different type carbon nanostructures. For example, polythiophene (PT) [43,44] and polypyrrole (PPy) also react hypergolicly with fuming nitric acid to produce multi-coloured emissive carbon dots (Figure 9). Based on AFM studies, in both cases the dots appear nearly spherical in shape with narrow size distribution of 5–7 nm (Figure 9). In addition, they are dispersible in acetone giving bright photoluminescence upon UV irradiation (380 nm): the polythiophene-derived dots give red-emission (Figure 9a), whereas those from polypyrrole green-emission [45] (Figure 9b). The different emissive properties of the dots probably reflect the different effect of heteroatom doping (S- vs. N-doped dots). These results show the wider applicability of conductive polymers in the hypergolic synthesis of a variety of carbon nanomaterials. Tentatively, electron-withdrawing heteroatoms in close proximity to conjugated double bonds seem to favor ignition by fuming nitric acid (e.g., through the formation of energetic nitro-compounds or peroxide intermediates) [46,47].

**Figure 10:**
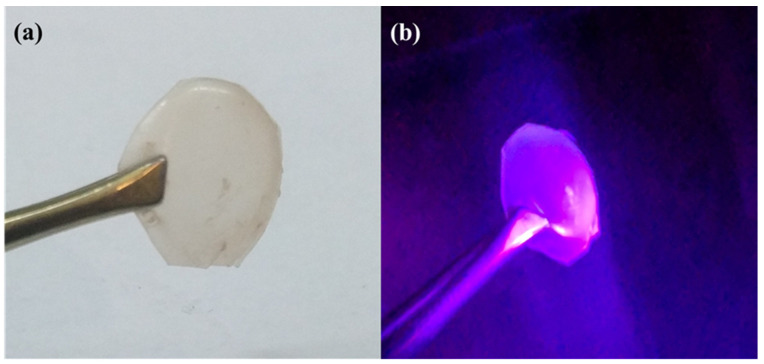
The red photoluminescent polymer nanocomposite containing polythiophene-derived dots under natural (**a**) and UV light (**b**).

The derived dots are further utilized to fabricate a photoluminescent polymer nanocomposite. A predetermined quantity of low polydispersity diblock copolymer polystyrene-*b*-polybutadiene (PS-*b*-PB) synthesized exclusively by anionic polymerization [48] was diluted in THF and simply mixed with a small amount of polythiophene-derived dots under stirring. The nanocomposite film was fabricated by casting and evaporating the solvent in a petri dish. The completely dried film was carefully taken off from the bottom of the dish to give a photoluminescent self-standing membrane (Figure 10).

## 3. Materials and Methods

For safety reasons all procedures were carried out in a fume hood with ceramic tiles bench using small amount of reagents.

### 3.1. Polyaniline Synthesis

**Scheme 1:**
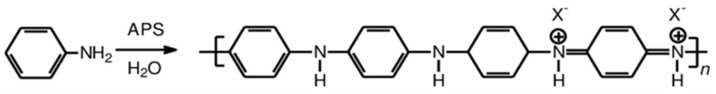
Molecular structure of polyaniline.

The emeraldine salt of polyaniline was synthesized by polymerization of aniline with ammonium persulfate (APS) as an oxidant and hydrochloric acid (HCl) (alternatively, commercial conductive polyaniline from Sigma–Aldrich can be used). In a typical procedure, a flask containing 9.313 g (0.1 mol) of distilled aniline (≥99%, Merck KGaA, Darmstadt, Germany) was dissolved in 100 mL of HCl 1 M (diluted from HCl 37%, Merck KGaA, Darmstadt, Germany) and kept at room temperature for 1 h under stirring. The solution of the oxidizing agent was prepared by dissolving 28.52 g (0.125 mol) of APS (≥98%, Merck KGaA, Darmstadt, Germany) in 52 mL of deionized water, which was prepared in another flask and was kept under ambient conditions for 1 h. The prepared solution was then dropwise added to the above prepared aniline solution for 1 h under continuous stirring. The synthesis was conducted in an ice bath at 0–2 °C. After the addition of APS, a dark green solution was obtained and was left under stirring for 24 h for completion of the polymerization. The solid was collected by filtration using a filter paper of 0.2 μm on a Büchner funnel followed by continuous rinsing with 1 M HCl/distilled water and acetone (99%, Sigma–Aldrich, St. Louis, MO, USA) at least 7–8 times until the solution became colorless. Finally, the product was solubilized in distilled water, sonicated for 1 h, added in acetone in order to precipitate the desired polymer which was then dried under vacuum at 50 °C in order to obtain the green powder form of polyaniline. The molecular structure of the emeraldine salt of polyaniline is shown in Scheme 1, where the counter ion X^-^ denotes acetate ions after treating the polymer with acetic acid.

### 3.2. Carbon Nanosheets

**Figure 11:**
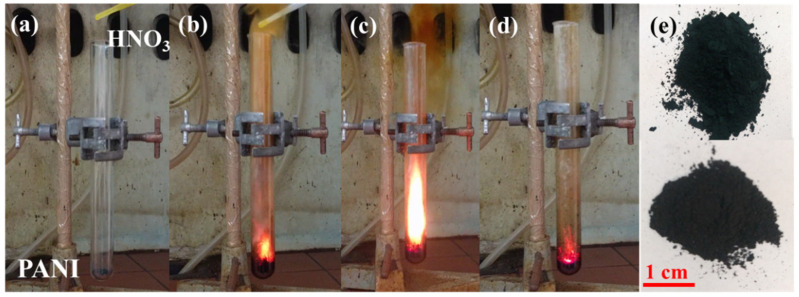
(**a**) The test tube containing polyaniline before nitric acid addition. (**b**–**d**) Dropwise addition of the acid sparked ignition, emitting an intense burst of flame. (**e**–**top**) The starting material used for the reaction; the dark green hue of polyaniline is indicative of its conductive state. (**e**–**bottom**) The residue within the tube was collected and washed extensively to afford a fine dark-brown powder.

One hundred mg of conductive polyaniline was placed into a glass test tube (diameter: 1.5 cm; length: 15 cm) followed by the slow, dropwise addition of 0.25 mL fuming nitric acid (100%, Sigma–Aldrich, St. Louis, MO, USA). The two reagents ignited with a short delay upon contact to form a carbon remnant within the test tube. The energy released from the reaction probably causes the in situ carbonization of polyaniline. The residue was collected and washed abundantly with de-ionized water, ethanol (≥99% Merck KGaA, Darmstadt, Germany), *N*-Methyl-2-pyrrolidone (NMP, ≥99% Merck KGaA, Darmstadt, Germany) and acetone (≥99% Merck KGaA, Darmstadt, Germany) prior drying. A fine carbon powder was obtained at yield 5% (N_2_ specific surface area: 146 m^2^ g^−1^). The synthetic procedure is illustrated in Figure 11. The reaction was consecutively conducted in several test tubes in order to collect enough material for characterizations.

### 3.3. Characterization Techniques

The powder X-ray diffraction patterns were collected on a D8 Advanced Bruker diffractometer (Bruker, Billerica, MA, USA) using CuKα (40 kV, 40 mA) radiation and a secondary beam graphite monochromator. The patterns were recorded in a 2-theta (2θ) range from 2 to 80°, in steps of 0.02° and counting time 2 s per step. Raman spectra were obtained on a DXR Raman microscope using a laser excitation line at 455 nm, 2 mW (Thermo Scientific Waltham, MA, USA). Attenuated total reflection infrared spectroscopy (ATR-IR) measurements were performed using a Jasco IRT-5000 microscope coupled with an FT/IR-4100 spectrometer (Jasco, Easton, MD, USA). The ZnSe prism of the ATR objective had a 250 μm area in contact with the sample. Background was subtracted and the baseline was corrected for all spectra. UV-Visible (UV-Vis) spectra were measured in quartz cuvettes with a UV2401 (PC)-Shimadzu spectrophotometer (Shimadzu, Kyoto, Japan). X-ray photoelectron spectroscopy (XPS) was carried out with a PHI VersaProbe II (Physical Electronics, Chanhassen, MN, USA) spectrometer using an Al-Kα source (15 kV, 50 W). The obtained data were evaluated with the MultiPak (Ulvac–PHI, Inc., Miami, FL, USA) software package. The spectral analysis included Shirley background subtraction and peak deconvolution employing mixed Gaussian-Lorentzian functions. Scanning electron microscopy (SEM) images were obtained using a JEOL JSM-6510 LV SEM Microscope (JEOL Ltd., Tokyo, Japan) equipped with an X–Act EDS-detector by Oxford Instruments, Abingdon, Oxfordshire, UK (10 kV). Suspensions of the material with a 0.1% *w/v* concentration were prepared and drop-casted onto carbon coated copper grids (CF300-CU-UL, carbon square mesh, CU, 300 mesh from Electron Microscopy Science, Hatfield, England). The drop-casted sections were studied using transmission electron microscopy TEM (JEOL 2010 TEM equipped with a LaB_6_ type emission gun operating at 160 kV, JEOL Ltd., Tokyo, Japan). Energy-dispersive X-ray spectroscopy (EDS) was recorded on an Oxford x-MAX 80T (SSD) at 200 kV accelerating voltage. Scanning transmission electron microscopy high-angle annular dark-field (HAADF) imaging analyses for EDS mapping of elemental distributions on the products were performed with an FEI Titan HRTEM microscope operating at 80 kV. Atomic force microscopy (AFM) images were collected in tapping mode with a Bruker Multimode 3D Nanoscope (Ted Pella Inc., Redding, CA, USA) using a microfabricated silicon cantilever type TAP-300G, with a tip radius of <10 nm and a force constant of approximately 20–75 N m^−1^.

## 4. Conclusions

Polyaniline and fuming nitric acid react hypergolicly upon contact to afford carbon nanosheets at ambient conditions in a fast and spontaneous manner. The structural and morphological characteristics of the produced carbon were evaluated with a large variety of techniques such as XRD, ATR/IR, Raman, UV-Vis, XPS and SEM/AFM/TEM, confirming the phylomorphous nature of the material as well as the incorporation of O, N heteroatoms in its structure (e.g., O, N–containing nanosheets). Liquid-phase exfoliation of the nanosheets in DMF produces a fine colloidal dispersion of single layers. The method can be extended beyond polyaniline to include other conductive polymers polythiophene and polypyrrole, hence creating even more nanostructures (e.g., photoluminescent carbon dots). The present systems build upon previous ones from our group on the hypergolic synthesis of carbon nanomaterials, demonstrating the universal character of this radically new preparative approach in materials science. Of particular importance is also the fact that the demonstrated reactions are new in the field of conductive polymers, thus further enriching their chemistry and reactivity with another paradigm in point. Lastly, the discussed pairs broaden the horizons of hypergolic rocket propellants with new solid polymer fuels.

## Data Availability

The data presented in this study are available on request from the corresponding author.

## Sample Availability

Not available.
